# Genome-Wide Identification and Expression Analysis of *C3H Zinc Finger* Family in Potato (*Solanum tuberosum* L.)

**DOI:** 10.3390/ijms241612888

**Published:** 2023-08-17

**Authors:** Zeyi Deng, Zhijiang Yang, Xinyan Liu, Xiumei Dai, Jiankui Zhang, Kexuan Deng

**Affiliations:** 1 College of Agronomy and Biotechnology, Southwest University, Chongqing 400715, China; swudzy@email.swu.edu.cn (Z.D.); yzj137019@email.swu.edu.cn (Z.Y.); liuxinyan@email.swu.edu.cn (X.L.); dxm72031@swu.edu.cn (X.D.); jkzhang@swu.edu.cn (J.Z.); 2 Engineering Research Center of South Upland Agriculture, Ministry of Education, Chongqing 400715, China

**Keywords:** potato, *C3H* gene family, bioinformatics, tuber development, heat stress

## Abstract

Transcription factors containing a CCCH structure (C3H) play important roles in plant growth and development, and their stress response, but research on the *C3H* gene family in potato has not been reported yet. In this study, we used bioinformatics to identify 50 *C3H* genes in potato and named them *StC3H-1* to *StC3H-50* according to their location on chromosomes, and we analyzed their physical and chemical properties, chromosome location, phylogenetic relationship, gene structure, collinearity relationship, and cis-regulatory element. The gene expression pattern analysis showed that many *StC3H* genes are involved in potato growth and development, and their response to diverse environmental stresses. Furthermore, RT-qPCR data showed that the expression of many *StC3H* genes was induced by high temperatures, indicating that *StC3H* genes may play important roles in potato response to heat stress. In addition, Some *StC3H* genes were predominantly expressed in the stolon and developing tubers, suggesting that these *StC3H* genes may be involved in the regulation of tuber development. Together, these results provide new information on *StC3H* genes and will be helpful for further revealing the function of *StC3H* genes in the heat stress response and tuber development in potato.

## 1. Introduction

Transcription factors play key roles in regulating multiple important physiological processes in plants. Studying the functional characteristics of transcription factors is crucial to revealing their biological processes [1,2]. Zinc finger proteins are sequence-specific transcription factors, which are generally considered to bind DNA or interact with protein to regulate gene expression. However, some types of zinc finger proteins, such as the C3H protein, which accounts for about 0.8% of the whole zinc finger family, have specific biological functions [3,4,5,6]. The C3H zinc finger proteins of plants have one to six copies of the conserved C3H motif (consisting of three Cys and one His). Based on the different numbers of amino acid spacers between Cys and His in the C3H motif, the common sequence of the C3H motif was initially defined as C-X_6-14_-C-X_4-5_-C-X_3_-H (X represents any amino acid) [7]. After a series of studies, the C3H motifs were defined as C-X_4–17_-C-X_4–6_-C-X_3_-H [8].

Based on the number and distribution of C3H motifs, C3H zinc finger proteins are divided into two types: the tandem C3H-type zinc finger (TZF) and the non-TZF proteins [9]. TZF proteins only contain two tandem C3H-type zinc finger motifs, whereas non-TZF proteins have one or more than two C3H-type zinc finger motifs [10]. In plants, the TZFs with C3H motifs also are called RR-TZFs [11]. Recent studies have shown that C3H proteins are involved in plant development and their stress response [12,13]. *OsC3H10*, a TZF gene in rice, has been shown to participate in the regulation of drought resistance by increasing the expression of stress-related genes [14], and Jamil et al. also found that the *OsC3H33*, *OsC3H37*, and *OsC3H50* genes are induced by salt stress [15]. Boyi et al. found that the RR-TZF genes in *Brassica napus* are involved in ABA or the drought stress response [9]. Seok et al. found that the non-TZF gene *AtC3H17*, as a nuclear transcriptional activator, promoted seed germination and seedling development, and caused early flowering by directly activating the transcription of *OLEO1*, *OLEO2*, and *CRU3* [16]. *Arabidopsis* TZF1 (AtTZF1) plays an important role in the sugar response and maintaining a normal ABA/GA response [17]. AtC3H59/ZFWD3 plays an essential role in seedling development and seed germination and development by interacting with the PPPDE gene family protein [18]. C3H zinc finger protein OsGZF1 affects glutelin accumulation during seed development [19]. The overexpression of C3H zinc finger protein *GHZFP1* from cotton could enhance the tolerance to salt stress and fungal diseases in tobacco [20]. The maize zinc finger protein *ZmC3H54* is strongly induced by ABA treatment and drought stress, which may play an important role in coping with abiotic stress [21,22].

Potato is the fourth-largest major crop in the world and an irreplaceable and important component of the human diet in some countries [23]. The importance of potatoes in securing food and nutritional security has been identified by the Food and Agriculture Organization (FAO) of the United Nations [24]. The tubers of potatoes are the main economic part due to their calorific and nutritional value [25]. Potato tuber development and growth is mainly the continuous development of the underground stolon. During this process, the top of the stolon (S1) expands and gradually grasps to become a tuber (S2–S6). Afterward, the top of the stolon continues to expand and gradually forms a mature tuber [26]. The formation of potato tubers is influenced by various environmental factors, among which high temperature is one of the most impactful environmental stresses. In addition, the growth and development of potatoes are also influenced by hormones. Potato planting is facing increasingly serious problems and leading to a decline in their yield and quality [27,28]. Therefore, revealing the response mechanism of potatoes to different stresses and breeding new varieties with high quality and high yield has become an important goal and challenge in potato research.

The *C3H* transcription factor family is known to be essential in the regulation of plant development and stress tolerance [12,14,29,30,31,32]. However, current research on the function of C3H zinc finger proteins in potato is still limited. In this study, we identified and systematically analyzed the *C3H* gene family in potato by using bioinformatics methods, and we investigated the expression profile of *StC3H* genes in different tissues and under different environmental stress based on transcriptome data. Moreover, we performed RT-qPCR to examine the *StC3H* genes’ response to high-temperature stress and the expression pattern of *StC3H* genes in the potato seedling and tuberization stages. Our data provide important information about the *C3H* gene family in potato and lay an important foundation for further functional studies.

## 2. Results

### 2.1. Identification of C3H Gene Family in Potato

**Figure 1:**
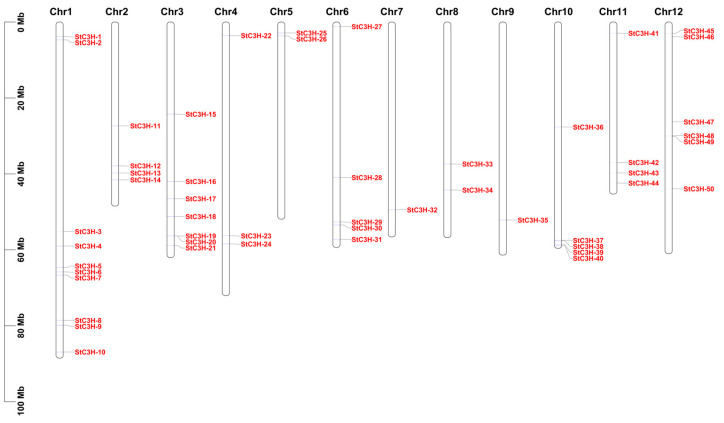
Chromosomal locations of StC3H proteins. Red labels represent the name of the potato C3H genes.

In this study, 50 members of the C3H family were obtained from the potato genome by using the HMMER 3.0 software according to the C3H domain (PF00642). A total of 50 potato *C3H* genes could be mapped on chromosomes and were renamed *StC3H-1* to *StC3H-50*, based on their order on the chromosomes (Figure 1). Among these C3H proteins, the number of amino acids was between 129 and 858, with the predicted molecular weight ranging from 14.56 to 96.13 kDa, and the average molecular weight of StC3H proteins was 50.96 kDa. Although the length of some StC3H proteins was less than 300 amino acids (10 StC3H proteins), most StC3H proteins were over 300 amino acids long (40 StC3H proteins). The pI values ranged from 5.14 to 9.62. The grand average of hydropathicity ranged from −0.315 to −1.540, indicating that all StC3H proteins were hydrophilic proteins (Table 1). According to the rule that an instability coefficient below 40 indicates a stable protein and isoelectric point below 7 indicates an acidic protein, it was inferred that 30 members were acidic proteins and 20 members were basic proteins; only 7 members were stable proteins, and most members were unstable proteins.

Consistent with existing research, the subcellular localization results showed that most of the StC3H proteins were located in the nucleus, suggesting that most StC3H proteins functioned in the nucleus. Meanwhile, some StC3H proteins were located in the cytoplasm, chloroplast, peroxisome, or cytoskeleton, implying that some StC3H proteins had special functions in the cell (Table 1) [13,33,34,35,36].

### 2.2. Location Analysis of C3H Gene Family Members on the Chromosome in Potato

As shown in Figure 1, *StC3H* genes were unevenly distributed on 12 potato chromosomes. Chromosome 1 contained the largest number of *StC3H* genes, with a total of 10 genes, while chromosome 7 and chromosome 9 contained only one *StC3H* gene. A chromosome region containing two or more genes within 200 kb could be defined as a tandem duplication event [37]. We found three pairs of *StC3H* genes located on potato chromosomes within 200 kb, including *StC3H-19* and *StC3H-20*, *StC3H-37* and *StC3H-38*, and *SlC3H-48* and *SlC3H-49*. To further identify tandem duplication events in these genes, the Multiple Collinearity Scan toolkit (MCScanX) in TBtools was used, and data showed no tandem duplication events in *StC3H* gene family [38,39], suggesting that the evolution of *StC3H-19* and *StC3H-20*, *StC3H-37* and *StC3H-38*, and *SlC3H-48* and *SlC3H-49* did not exhibit tandem duplication.

### 2.3. Multiple Sequence Alignment and Phylogenetic Analysis of C3H Gene Family in Potato

**Figure 2:**
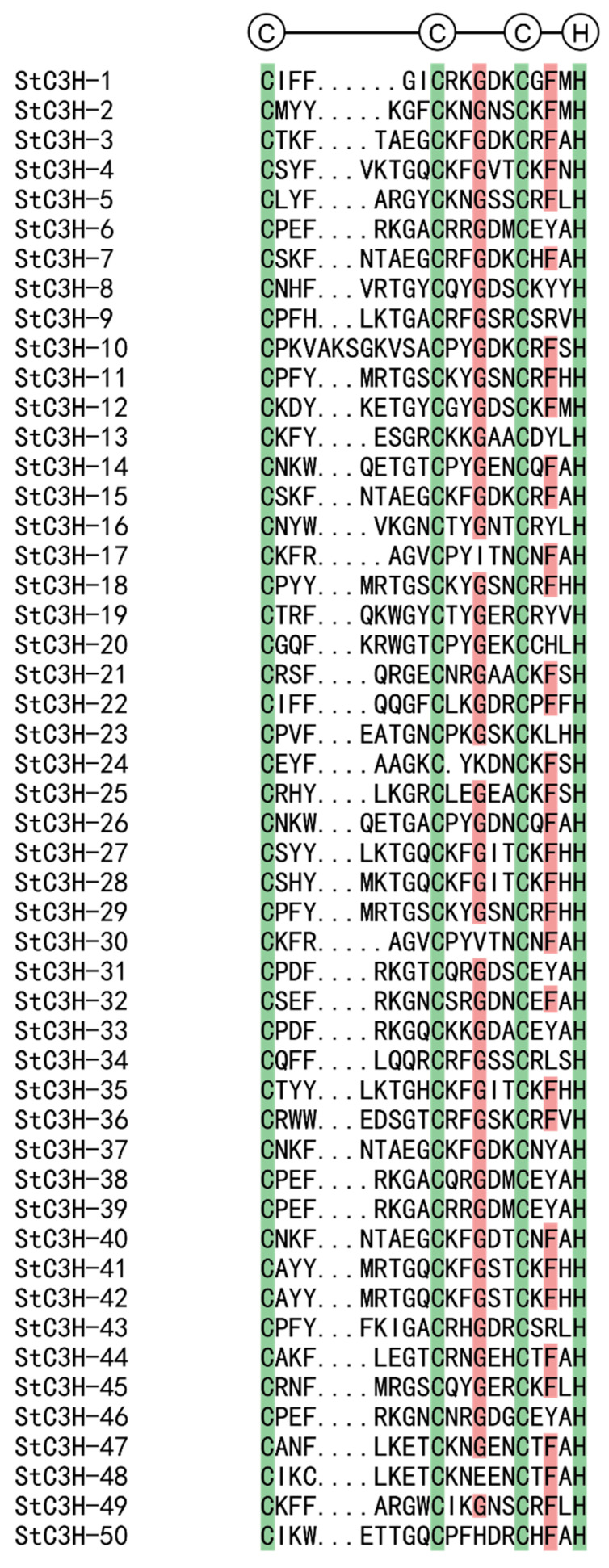
Amino acid sequence alignments of potato C3H proteins; green and brown shading indicate identical and conserved amino acid residues, respectively. The three Cys and one His residues putatively responsible for the C3H zinc finger structure are indicated.

To further clarify the sequence characteristics of *StC3H* gene family members, we compared potato C3H protein sequences. Since multiple motifs in the same protein were usually similar and had miscellaneous functions, we found one C3H motif of each protein for analysis (Figure 2). The results showed that all the 50 StC3H protein sequences contained C3H motifs, but the amino acid space and quantity of C3H residues were different, which may have been one of the reasons for the specificity function of different C3H members. It is worth mentioning that there are two abundant C3H motifs, C-X_7_-C-X_5_-C-X_3_-H and C-X_8_-C-X_5_-C-X_3_-H, in C3H proteins. In all 50 StC3H proteins, 20 StC3H proteins (40%) contained the C-X_7_-C-X_5_-C-X_3_-H motif and 24 StC3H proteins (48%) contained the C-X_8_-C-X_5_-C-X_3_-H motif, indicating that these two types of C3H motif may have been the ancestors of other C3H genes, which is consistent with the results obtained from plants such as *Arabidopsis thaliana*, rice, and corn, and animals such as mice [21,40]. We also found that some rarely reported C3H motifs exist in the StC3H family, such as C-X_11_-C-X_5_-C-X_3_-H and C-X_7_-C-X_4_-C-X_3_-H. In the C3H motifs, 45 StC3H proteins contained a glycine residue between the second and third cysteine residues, and 34 StC3H proteins contained phenylalanine residues between the third cysteine and histidine residues, reflecting their high conservation across the StC3H protein family (Figure 2). In addition, most proteins had an aromatic amino acid residue in the second or third position after the first cysteine residue.

**Figure 3:**
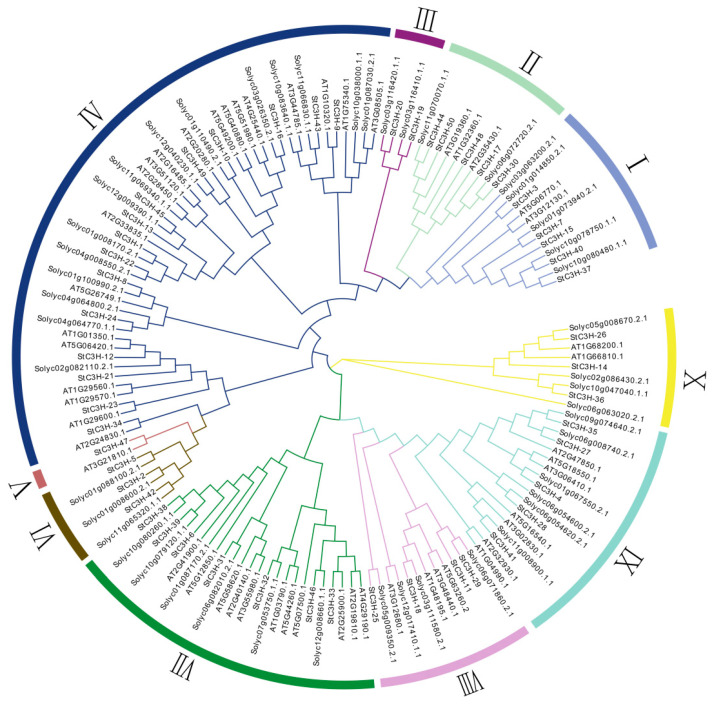
Phylogenetic tree of *C3H* genes in potato, *Arabidopsis*, and tomato. The differently colored arcs indicate different groups. The 159 lengths of the consensus amino acid sequences were used to construct the phylogenetic trees. The groups were named from ‘I’ to ‘X’. Genes with the prefix ‘St’ indicate the potato *C3H* gene, genes with the prefix ‘AT’ indicate the *Arabidopsis C3H* gene, and genes with the prefix ‘Solyc’ indicate the tomato C3H gene.

Furthermore, we constructed a phylogenetic tree using the consensus amino acid sequence of each member of potato, *Arabidopsis* and tomato to explore the evolutionary relationships among *C3H* zinc finger genes (Figure 3 and Figure S1), and the full-length and consensus amino acid sequence of all C3H protein was list in Table S1. As shown in Figure 3, StC3H family could be divided into 10 groups (I, II, III, IV, V, VI, VII, VIII, IX, X). The number of StC3H proteins was uneven. Group IV was the largest (15 StC3H proteins), followed by Group VII (7 StC3H proteins) and Group I, II, and IX (each with 5 StC3H proteins). Group III, V, VI, VIII and X have 2, 1, 3, 4 and 3 StC3H proteins, respectively.

### 2.4. Analysis of Gene and Protein Structure and Function Prection of C3H Family in Potato

By analyzing the gene structure of *StC3H* genes, we found that *C3H* genes had 1 to 10 CDS (coding sequence) regions (Figure S2). *StC3H-19*, *StC3H-20*, *StC3H-11*, *StC3H-43*, *StC3H-48*, *StC3H-50*, *StC3H-47*, and *StC3H-8* did not have an UTR (untranslated region), and other members of the C3H gene family all had UTRs. In addition, the intron was not enriched in the *StC3H* gene family. Some StC3H genes did not have introns, including *StC3H-3*, *StC3H-46*, *StC3H-6*, *StC3H-39*, *StC3H-38*, *StC3H-32*, *StC3H-31*, and *StC3H-33*, while *StC3H-10* and *StC3H-45* had nine introns.

**Figure 4:**
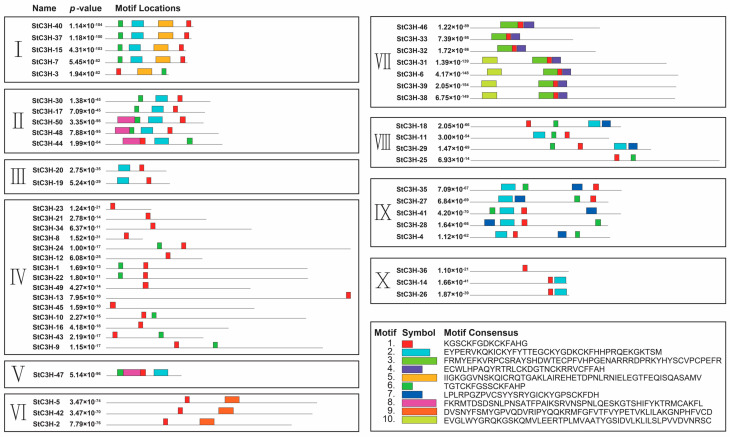
Conserved motif of StC3H protein. The colorful boxes delineate different motifs. The clustering was performed according to the results of the phylogenetic analysis. I–X means the ten subfamilies of StC3H proteins.

To further investigate the characteristic regions of proteins, we used the online MEME suite program to identify the conserved motifs of the C3H proteins in potato. Ten conserved motifs were detected, ranging from 6 to 50 amino acids in length (Figure 4; Table S2). Motif 1 exists in all StC3H proteins. Unsurprisingly, similar conserved motifs were found within the same groups. Motif 7 was widely present in groups VIII and IX, motif 5 was only present in group I, motif 9 was only present in group VI, and motif 3 and motif 4 were only found in group VII. In addition, members within the same group also exhibited some difference in their conserved motifs. In group VII, StC3H-31, StC3H-6, StC3H-39, and StC3H-38 had motif 10, while StC3H-46, StC3H-33, and StC3H-32 did not have motif 10. Most genes in the same branch had similar conserved motif compositions and structures, suggesting that they were functionally similar.

Furthermore, the prediction of protein structure and protein–DNA/RNA interaction data showed that StC3H proteins had different three-dimensional structures, and all StC3H proteins with motif 2 or motif 6 in groups I, II, III, V, and IX could interact with RNA, indicating that the presence of motif 2 or motif 6 may result in an interaction between StC3H proteins and RNA. In addition, we found that StC3H-6, StC3H-31, and StC3H-39 proteins with motif 1, motif 3, motif 4, and motif 10 could interact with DNA or RNA. However, unlike StC3H-6, StC3H-31, and StC3H-39, StC3H-38 containing motif 1, motif 3, motif 4, and motif 10 could not interact with DNA or RNA. Similarly, StC3H-46, StC3H-33, and StC3H-32 contained motif 1, motif 3, and motif 4, but only StC3H-46 and StC3H-32 could interact with RNA (Table S3). These data suggest that motif 1, motif 3, motif 4, or motif 10 were not sufficient to determine the interaction between StC3H and DNA/RNA. Meanwhile, the results of protein structure prediction showed that StC3H proteins in group VII had significant differences in protein structure between StC3H proteins with or without motif 10, implying that motif 10 may impact the structure of StC3H proteins (Figure S3).

### 2.5. Collinearity Analysis of C3H Gene Family

**Figure 5:**
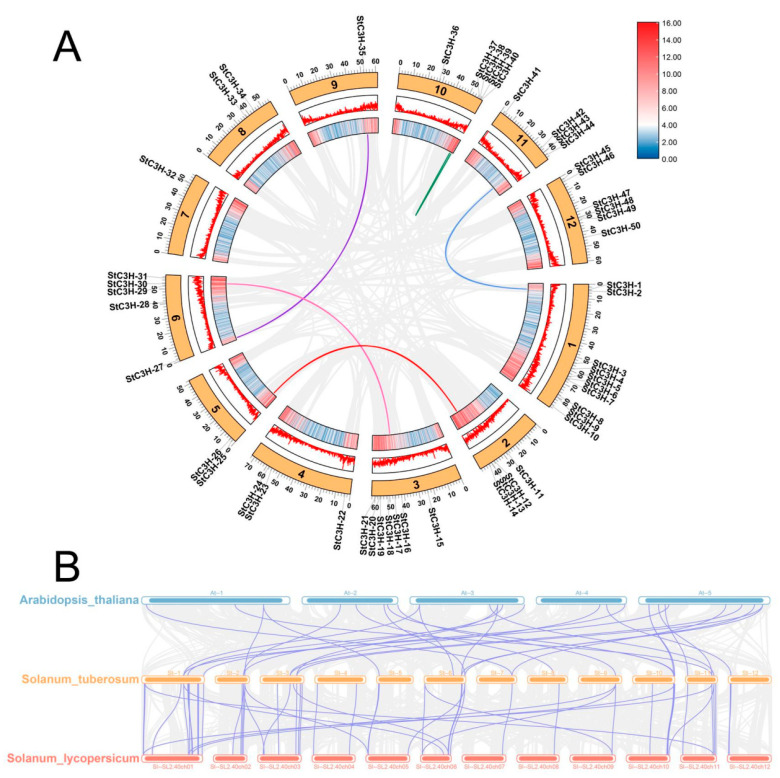
Collinearity of *StC3H* genes and interspecific collinearity of *C3H* genes. (**A**) Gray lines indicate all synteny blocks in the potato genome and colored lines indicate duplicated *C3H* gene pairs. (**B**) Chromosome number is indicated at the middle of each chromosome. Synteny analysis of *C3H* genes among potato, *Arabidopsis*, and tomato. Gray lines indicate the collinear bars within the potato and other plant genomes, whereas the purple lines highlight the syntenic *C3H* gene pairs.

Gene duplication plays a crucial role in the process of biological evolution [41]. To explore the possible relationship and the potential gene duplication in the *StC3H* family, we used the TBtools v1.120 to analyze the collinearity of *StC3H* gene family members. As shown in Figure 5A, among the members of the *StC3H* gene family, *StC3H-2* and *StC3H-42*, *StC3H-14* and *StC3H-26*, *StC3H-17* and *StC3H-30*, *StC3H-27* and *StC3H-35*, and *StC3H-37* and *StC3H-40* were collinear, indicating that some *StC3H* genes were possibly generated by gene duplication and the segmental duplication events played a major driving force in *StC3H* evolution.

To investigate the origin and development of the *C3H* gene family in potato, we used TBtools to analyze the collinearity of *C3H* genes in potato, *Arabidopsis*, and tomato. The results showed that there were 26 pairs of collinearity between potato and *Arabidopsis*, and 48 pairs of collinearity between potato and tomato (Figure 5B). Among them, potato and tomato belonged to different crops in the same genus, so their collinearity was better than that of potato and *Arabidopsis*. The comparison and analysis of *C3H* genes between potato and other plants are helpful for establishing interspecific relationships and predicting gene functions.

### 2.6. Cis-Acting Elements Analysis

**Figure 6:**
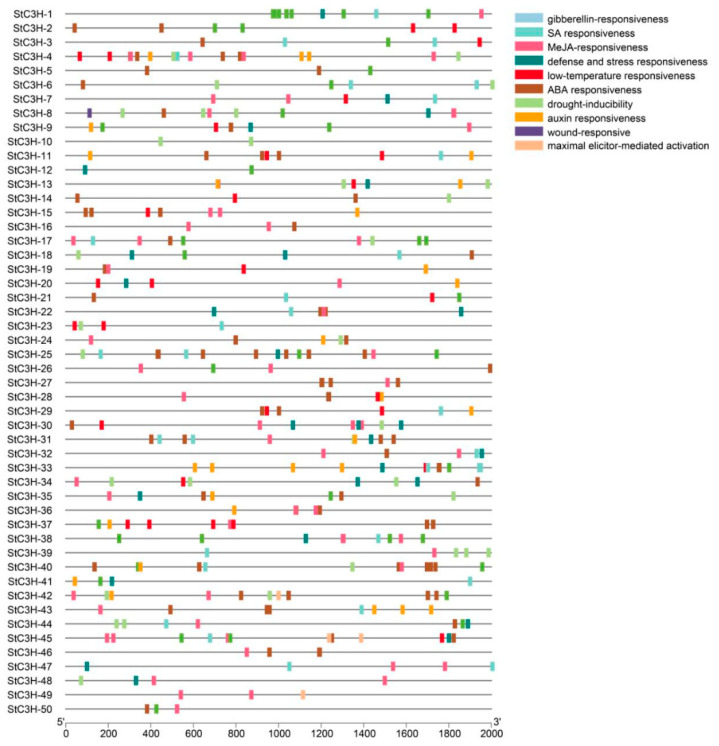
Cis-acting elements in promoter region of *StC3H* genes.

To study the potential regulatory mechanism of *StC3H* family members, we analyzed the cis-regulatory element of the 2000 bp sequence upstream of *StC3H* genes (Figure 6). The results showed that some basic conserved motifs, such as TATA-box and CAAT-box, existed in the promoter region of *StC3H* genes. In addition, the promoter region of *StC3H* family genes also had many important cis-regulatory elements related to stress, hormones, and plant growth and development with high frequency, including auxin responsiveness (29), abscisic acid responsiveness (100), gibberellin responsiveness (39), methyl jasmonate responsiveness (120), salicylic acid responsiveness (31), defense and stress responsiveness (27), drought inducibility (32), and low temperature responsiveness (29).

### 2.7. Expression Analysis of C3H Gene Family in Potato

**Figure 7:**
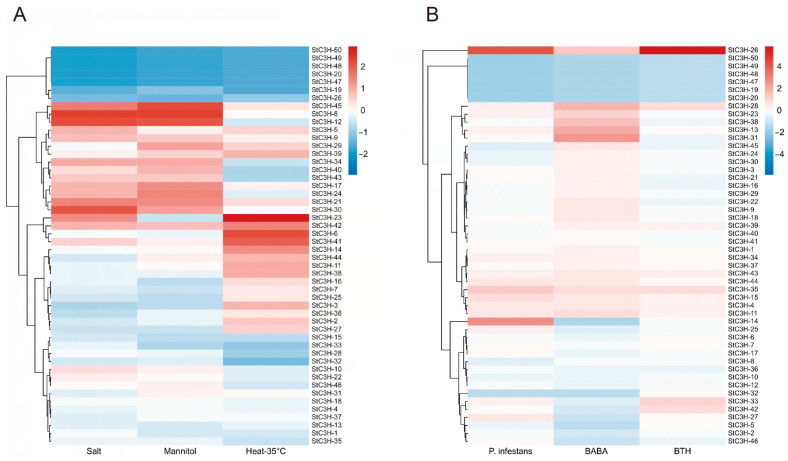
Heatmap of *StC3H* genes in response to environment stress in potato. (**A**) Expression profile of 50 *StC3H* genes under salt stress, mannitol stress, and heat stress treatment. (**B**) Expression profile of 50 *StC3H* genes under *P. infestans*, BABA, and BTH treatment.

To study the response of the *StC3H* gene family to biotic and abiotic stresses, we obtained transcriptome data from the potato genome database. The expression levels of *StC3H* genes under different stresses, such as salt, mannitol, heat, *P. infestans*, β-aminobutyric acid (BABA), and benzothiadiazole (BTH) were illustrated (Figure 7). The results of the *StC3H* gene expression level under abiotic stress are shown in Figure 7A; we found that *StC3H-45*, *StC3H-8*, and *StC3H-12* had strong responses to salt and drought, with significantly increased expression levels. Some *StC3H* genes had a significant response to salinity and drought, but had no response to high temperatures, such as *StC3H-5, StC3H-9*, and *StC3H-30. StC3H-42* was up-regulated under salt, drought, and high-temperature stress. Some *StC3H* genes had a response to high temperatures stress, but had no response to salt and drought stress, such as *StC3H-6*, *StC3H-41*, *StC3H-14*, *StC3H44*, etc. *StC3H-50*, *StC3H-49*, *StC3H-48*, *StC3H-20*, *StC3H-47*, *StC3H-19*, and *StC3H-26* were strongly inhibited by salt, drought, and high-temperature stress; other genes did not show significant changes in response to these stresses. The results of the *StC3H* gene expression level under biotic stress or immune inducers (β-aminobutyric acid (BABA) and benzothiadiazole (BTH)) are shown in Figure 7B. *StC3H-26* was up-regulated by *P. infestans*, BABA, and BTH, especially under BABA and BTH treatment. *StC3H-50*, *StC3H-49*, *StC3H-48*, *StC3H-47*, *StC3H-19*, and *StC3H-20* were down-regulated by *P. infestans*, BABA, and BTH. *StC3H-28*, *StC3H-23*, *StC3H-38*, *StC3H-13*, and *StC3H-31* were up-regulated under BABA treatment, but poorly responded to *P. infestans* and BABA. *StC3H-14* was strongly upregulated by *P. infestans* and significantly down-regulated by BABA, and no response to BTH treatment.

**Figure 8:**
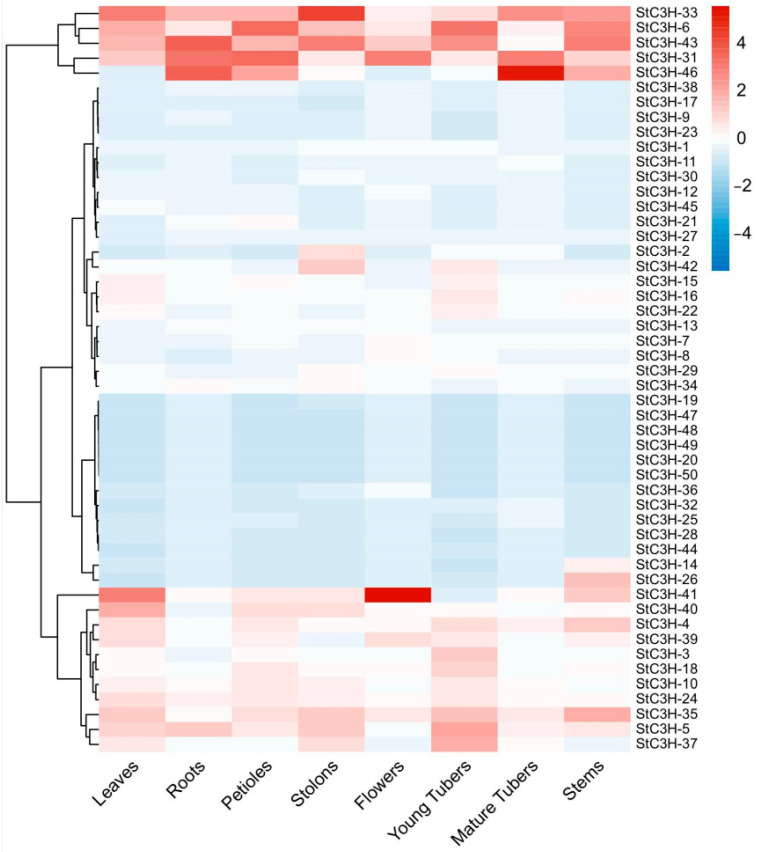
Tissue-specific expression analysis of *StC3H* genes. Expression profile of *StC3H* genes in leaves, roots, petioles, stolons, flowers, young tubes, material tubes, and stems.

To study the role of *StC3H* genes in growth and development, we obtained transcriptome data from the potato genome database, and the expression levels of *StC3H* genes in different tissues are displayed in Figure 8. *StC3H-33*, *StC3H-6*, *StC3H-43*, and *StC3H-31* were expressed in all tested tissues, and *StC3H-33* was highly expressed in the stolon. *StC3H-46* was expressed in the roots, petioles, mature tubers, and stems, and highly expressed in mature tubers. *StC3H-41* was highly expressed in flowers. *StC3H-4*, *StC3H-39*, *StC3H-35*, and *StC3H-5* were expressed in the leaves, petioles, young tubers, and stems. The expression levels of other members, i.e., *StC3H-40*, *StC3H-3*, *StC3H-18*, *StC3H-10*, *StC3H-24*, and *StC3H-37*, were very low. These results show that many *StC3H* genes were specifically and highly expressed in some tissues, indicating that *StC3H* genes had different functions in potato.

### 2.8. RT-qPCR Verification of Expression Level of StC3H Gene Family in Potato

**Figure 9:**
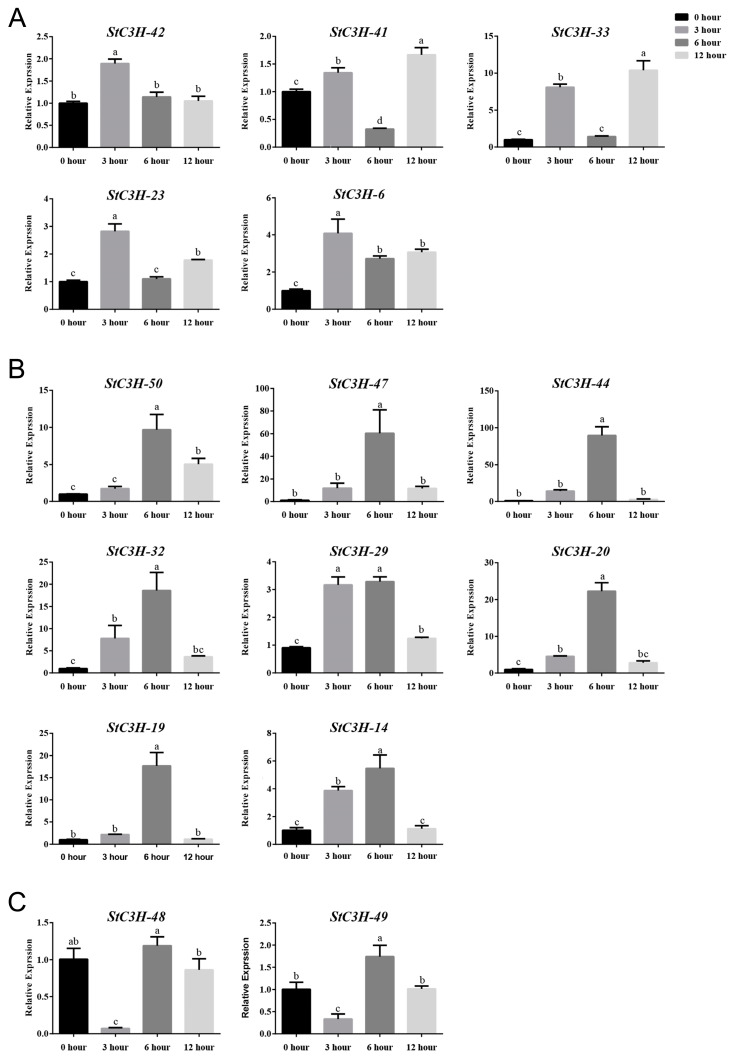
RT-qPCR analyses of differential expression of 15 *StC3H* genes under heat stress treatment. Based on the expression pattern of *StC3H* genes under heat stress, we classified them into three categories: (**A**) gene expression level increased after 3 h of treatment, then decreased after 6 h of treatment, and increased at 12 h of treatment, (**B**) gene expression level continued to increase within 3 h and 6 h of treatment, and then decreased after 12 h of treatment, and (**C**) gene expression level decreased after 3 h of treatment, and then increased after 6 h and 12 h of treatment. Error bars represent standard deviations of the means from three independent experiments. Different lowercase letters indicate significant differences (*p* < 0.05).

Heat stress negatively affects the growth and development of plants, leading to reductions in productivity and yield [25]. Temperature is one of the key uncontrollable factors that affect the tuberization of potato, and significantly affects potato yield [42]. We conducted RT-qPCR to analyze *StC3H* genes that responded to heat stress (Figure 9). The results showed that the expression of *StC3H-6*, *StC3H-23*, and *StC3H-42* significantly increased after 3 h of high-temperature treatment, and then decreased. The expression levels of *StC3H-33* and *StC3H-41* significantly decreased after 6 h of high-temperature treatment, but reached the highest expression levels after 12 h of treatment. With the extension of the high-temperature treatment time, the overall expression levels of *StC3H-14*, *StC3H-19*, *StC3H-20*, *StC3H-29*, *StC3H-32*, *StC3H-44*, *StC3H-47*, and *StC3H-50* showed a trend of first increasing and then decreasing, and they had the highest expression levels after 6 h of treatment. After 3 h of high-temperature treatment, the expression levels of *StC3H-48* and *StC3H-49* showed a sudden decrease, and then the expressions of *StC3H-48* and *StC3H-49* increased. These results indicate that *StC3H* may be crucial for potato’s response to heat stress.

**Figure 10:**
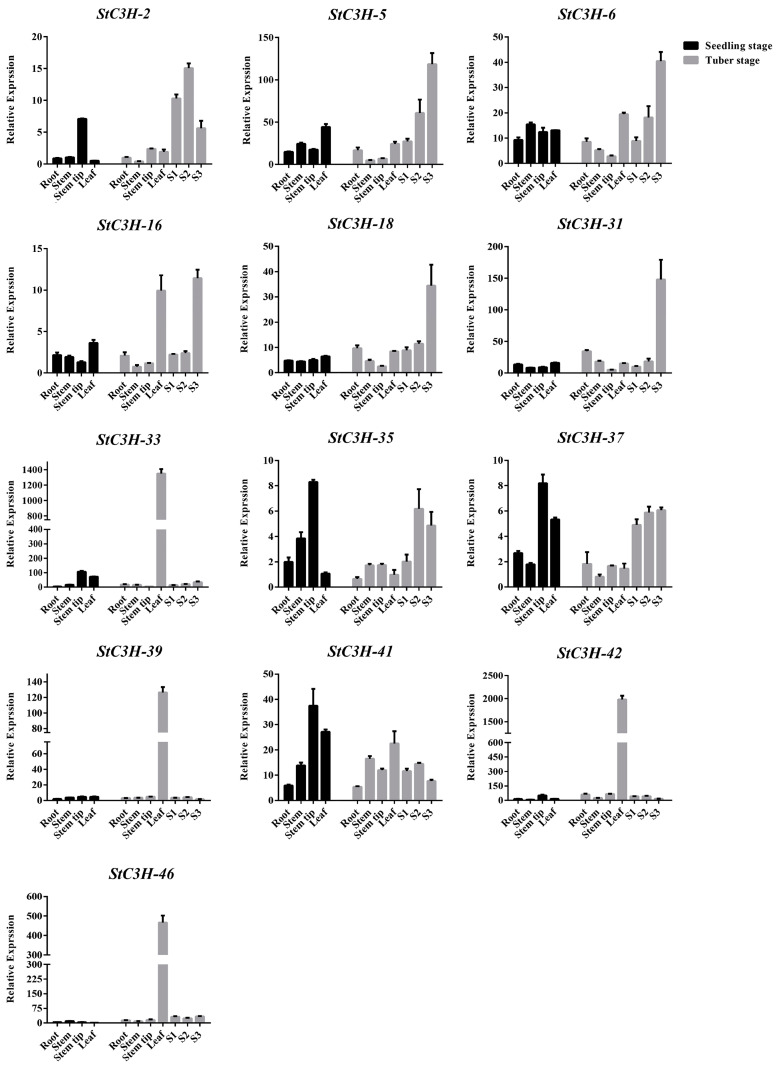
Relative expression of *StC3H* in different tissues in potato seedling stage and tuberization stage. Error bars represent standard deviations of the means from three independent experiments. S1 represents potato stolons. S2–S3 represent early stages of developing potato tubers [26].

In order to analyze the expression of the *StC3H* gene family in different potato tissues at the seedling and tuberization stages, we further analyzed the expression of 13 *StC3H* genes in potato tissues by RT-qPCR (Figure 10). The results showed that *StC3H-5*, *StC3H-6*, *StC3H-18*, and *StC3H-31* had the highest expression levels in S3, which may have played an important role in the development of tubers. *StC3H-2*, *StC3H-35*, *StC3H-37*, and *StC3H-41* were expressed in various tissues during the tuberization stage, and especially highly expressed in S1, S2, and S3. In addition, they were highly expressed at the stem tips during the seedling stage, indicating that these genes may have been closely related to the development of stem tips during the seedling stage and the development of tubers during the tuberization stage, respectively. The expression level of *StC3H-16* was high in leaves and S3. *StC3H-33*, *StC3H-39*, *StC3H-42*, and *StC3H-46* were specifically expressed in the leaves during the tuberization stage, indicating that they may have played a role in regulating the function of leaves during the tuberization stage. These results demonstrate that *StC3H* genes may have different functions in the different growth and development stages of potato.

## 3. Discussion

Zinc finger protein, as an important transcription factor family, plays crucial roles in cell differentiation, embryonic development, the regulation of gene transcription, and participation in protein–protein interactions [43,44,45]. Compared with other zinc fingers, such as the C2H2-type or CCCC-type gene families, there have been few studies on the *C3H* gene family. To date, some *C3H* gene families have been identified in *Arabidopsis*, *Solanum lycopersicum*, *Capsicum annuum*, *Hordeum vulgare*, *Pyrus betulaefolia*, *Dimocarpus longan Lour*, and other species [6,21,46,47,48,49,50,51,52]. And the functional study of *C3H* genes also indicates that the *C3H* family plays important roles in various aspects of plants, including plant growth and development and stress tolerance [13]. So far, detailed information on the *C3H* family in potato has not been uncovered. In this study, we identified and characterized 50 *C3H* genes from the potato genome, which were similar to those identified in other *Solanaceae* species, *Solanum lycopersicum* (47 members) and *Capsicum annuum* (57 members) [47,53]. The number of *C3H* genes varies among plant species, for example, rose (31 members) and *Medicago truncatula* (34 members) have fewer *C3H* members, and the number of *C3H* genes in *Brassica rapa* (103 members) and *Glycine max* (116 members) are significantly higher than those in other species [47,52,54,55,56]. Gene duplication is essential for the adaptation and evolution of organisms, and segmental and tandem duplications are considered to play important roles in the expansion of plant gene families [57]. Gene duplication analyses showed that ten *StC3H* genes (five colinear gene pairs) experienced segmental duplication events, and no tandem duplications were found in the *StC3H* family (Figure 5A). Interestingly, we also did not find any tandem duplication events within the *C3H* family in *Solanum lycopersicum* and *Capsicum annuum* by using MCScanX. Thus, our data suggest that segmental duplication is a primary contributor to the expansion of *StC3H* families, and the absence of tandem duplication events may reduce the number of *C3H* genes in *Solanaceae* species.

Comprehensive analysis of the *StC3H* gene family showed extensive variations in protein properties, phylogenetic relationships, conserved motifs, and protein structures, implying diverse and versatile functions of *StC3H* genes. The present study found that 50 identified *StC3H* genes encoded proteins varying from 129 to 858 amino acids, and St*C3H* proteins were mainly located in the nucleus, which is consistent with the *C3H* genes reported in *Arabidopsis*, rice, and other species (Table 1) [21,50]. But there are also some non-nuclear localized proteins, such as StC3H-37 in the cytoplasm and StC3H-40 in the chloroplasts. In a previous study, it was found that two cytoplasm-located proteins, TbZC3H12 and TbZC3H13, were required for optimal growth in Trypanosoma brucei, and some *C3H* proteins could shuttle between the nucleus and cytoplasm to modulate signal transduction and stress responses, suggesting that partial *StC3H* genes can function in different organelles of the cell [58,59,60]. Notably, we found that 10 StC3H proteins with fewer than 300 amino acids were not primarily located in the nucleus (4 in the cytoplasm, 4 in the nucleus, 1 in the cytoskeleton, and 1 in the chloroplasts), and only 4 StC3H proteins with more than 300 amino acids were not located in the nucleus (1 in the cytoskeleton, 1 in the peroxisome, and 2 in the chloroplasts) (Table 1). These data suggest that small StC3H proteins may have more different subcellular localizations, thereby modulating diversity functions in potato.

Conserved motif and structure analysis of the C3H proteins revealed that the C3H motifs were highly conserved, and the main present C3H motif in StC3H proteins was the C-X_7-8_-C-X_5_-C-X_3_-H-type C3H motif; 88% of StC3H proteins contained this kind of C3H motif (Figure 2). Although StC3H proteins had a conserved C-X_7-8_-C-X_5_-C-X_3_-H-type C3H motif, most members also had distinctive motifs (Figure 4). Phylogenetic tree and conservative motif analyses suggested that 50 *StC3H* genes could be divided into 10 groups, and the type and position of motifs varied among most groups; only motif 1 conservatively existed in all StC3H proteins (Figure 3 and Figure 4). However, DNA/RNA-binding function prediction and motif analysis showed that motif 1 could not determine whether StC3H proteins could bind to RNA or DNA. Interestingly, we found that all StC3H proteins containing motif 2 or motif 6 could interact with RNA, suggesting that motif 2 and motif 6 may be very important for the function of C3H proteins (Table S3).

As the binding sites of transcription factors, cis-regulatory elements in the promoter region determine the expression pattern of genes, thereby regulating various biological processes [61]. To provide insight into the functions of *StC3H* genes in potato, we analyzed the cis-regulatory elements and expression profiles of *StC3H* genes (Figure 6 and Figure 7). *StC3H-8*, *StC3H-12*, *StC3H-30*, and *StC3H-45*, which contained ABA-responsive elements, were highly expressed under salt and mannitol treatment. *StC3H-6*, *StC3H-23*, *StC3H-41*, and *StC3H-42*, with MeJA-responsive elements, were highly expressed under heat treatment. Notably, the expression pattern of *StC3H* genes was relatively consistent under drought and salt stress conditions, but it was significantly different from the results of heat treatment. These results indicate that *StC3H* genes may play regulatory roles in potato’s response to diversity stress, and potatoes may regulate the *StC3H* genes through the ABA and JA pathways to adapt to salt, mannitol, and heat stress. Unlike under abiotic stress, *StC3H* genes were not as sensitive to biotic stress, and only a few genes were significantly induced by abiotic stress, such as *StC3H-14*, *StC3H-26*, and *StC3H-31*, and these genes all had MeJA-responsive elements. Unexpectedly, we found that some *StC3H* genes with SA-responsive elements were not significantly induced under biotic stress, including *StC3H-17*, *StC3H-40*, *StC3H-48*, *StC3H-49*, etc. These results indicate that the *StC3H* family may primarily contribute to the abiotic stress response in potato, which is consistent with the findings for the role of *C3H* genes in other species [12,13]. In addition, we discovered that *StC3H-6*, *StC3H-31*, *StC3H-33*, *StC3H-41*, *StC3H-43*, and *StC3H-46* were highly expressed in the different tissues of potato, and most of these genes contained cis-elements responding to auxin and jasmonic acid (Figure 6 and Figure 8). Interestingly, these genes containing MeJA-responsive elements were not significantly up-regulated by diverse stresses, suggesting that auxin and JA signaling may be involved in regulating *StC3H* genes to modulate potato growth and development, and that potato may finely alter the JA signaling output, thereby regulating the expression of different *StC3H* genes and affecting the function of *StC3H* genes in stress tolerance and growth and development.

Potato is a major consumed food crop and is cultivated worldwide. However, potato yields are extremely affected by heat stress. Heat stress can significantly reduce potato yields by inhibiting potato growth and tuber development [62]. Recent studies have shown that *C3H* genes are involved in the response to heat stress. Xu et al. showed that the heterologous expression of HuTZF3 enhanced heat tolerance in transgenic *Arabidopsis* [63]. In pepper, some *C3H* genes were found to be significantly induced by heat stress, such as *PEPTY4* and *PEPTY51* [47]. Consistently, the expression profiles of *StC3H* genes showed that some *StC3H* genes were highly expressed under heat stress (Figure 7). In addition, the expression patterns of 15 candidate heat-induced genes were validated by RT-qPCR. The data showed that the expression of these *StC3H* genes was induced at different time points, suggesting that they may function at different stages of the potato response to heat stress. Meanwhile, we found that *StC3H* genes were significantly upregulated after 3 h of heat treatment containing many MeJA-responsive elements, implying that JA signaling may participate in the early heat stress response by regulating these *StC3H* genes in potato. As the storage organ of potato, the tuber is closely related to potato’s economic yield. Further understanding the tuberization process and identifying key genes are the aim of many plant biologists. In a previous study, it was found that StSP6A, a homolog of FLOWERING LOCUS T (FT), was generated in leaves, and then the StSP6A protein moved to the stolon to promote tuberization in potato [64]. Recent studies have identified that *C3H* genes participate in the regulation of flowering in different species [16,34,35,65,66,67,68]. For example, Wang et al. showed that the overexpression of *AaZFP3*, a CCCH-type ZFP from *Adonis amurensis*, could significantly induce the expression of *AtFT* to promote early flowering in *Arabidopsis* [65]. In addition, the knockdown of the expression of *AtTZF3* could induce early flowering in *Arabidopsis* [34]. Consistently, the overexpression of *AtTZ*F1, *AtTZF4*, *AtTZF5*, or *AtTZF6* could cause late flowering [17,69]. These data indicate that *C3H* genes have different functions in the regulation of plant flowering. In this study, StC3H-32, StC3H-33, and StC3H-46 shared the same branch as AtTZF_1_-6 in the phylogenetic trees (group VII), and RT-qPCR data showed that *StC3H-33* and *StC3H-46* were highly expressed in leaves during the tuberization stage, implying that *StC3H-33* and *St*C3H*-46* may regulate StSP6A or other flowering related genes to modulate tuberization or flowering in potato. Additionally, we found that *StC3H-32*, *StC3H-33*, and *StC3H-46* contained many ABA-responsive elements and MeJA-responsive elements in their promoter region, which is consistent with previous studies on AtTZFs. It has been shown that AtTZF1-6 is involved in the ABA and JA responses to modulate growth and stress response in *Arabidopsis* [17,34,69]. Interestingly, ABA and JA also play a significant role in the regulation of tuberization. Recently, it has been found that JA signaling is connected to potato tuberization, and the overexpression of an StJAZ1-like negative regulatory factor could restrict potato tuberization competence [26]. Jing et al. showed that StABI5-like 1, a transcription factor central to ABA signaling, could interact with StSP6A to promote tuberization [70]. Therefore, it can be speculated that *StC3H-32*, *StC3H-33*, and *StC3H-46* are involved in the ABA- and JA-mediated stress responses and tuberization in potato in different manners. Meanwhile, the result of RT-qPCR showed that the expressions of *St*C3H*-2*, *StC3H-5*, *StC3H-6*, *StC3H-16*, *StC3H-18*, *StC3H-31*, *StC3H-35*, and *StC3H-37* were significantly highly regulated in the stolon and developing tubers during the tuberization stage, and ABA-responsive elements and MeJA-responsive elements were also identified in these genes, especially in StC3H-6 (six MeJA-responsive elements), StC3H-35 (five MeJA-responsive elements and four ABA-responsive elements), and StC3H-37 (nine ABA-responsive elements). Even though it is unknown whether these highly expressed *StC3H* genes in the stolon and developing tuber are due to ABA or JA induction. These data suggest that these *StC3H* genes may be involved in regulating tuber development. And further studies may reveal whether there is a connection between these *StC3H* genes and hormones in regulating tuber development.

## 4. Materials and Methods

### 4.1. Plant Materials and Growth Conditions

The potato variety “Desiree” was used in this study. Four-week-old aseptic seedlings of Desiree in culture vessels were used to generate aseptic explants. Aseptic explants were subcultured on MS medium for 30 days, and then potato seedlings were planted in pots under 16 h of light/8 h of darkness at 22 °C in greenhouses.

To detect *C3H* gene expression in the seedling stage, potato seedlings were planted in pots for 1 week, and the roots, stems, leaves, and stem tips of seedlings were collected for RNA extraction. To detect *C3H* gene expression in the tuberization stage, potato seedlings were planted in pots for 4 weeks, and then grown under short daylight (8 h of light/16 h of darkness) for another 4 weeks to induce tuberization. And the roots, stems, leaves, stem tips, S1 (stolons), and S2–S3 (developing young tubers) were collected for RNA extraction.

For high-temperature treatment, potato seedlings were planted in pots for 1 week. And then, the potato seedlings were treated with a high temperature (37 °C), and seedlings were collected at 0 h, 3 h, 6 h, and 12 h for RNA extraction.

### 4.2. Identification of C3H Gene Family in Potato

We download a potato genome file, protein sequence file, and gff3 (general feature format Version 3) file from the Ensembl plants database (http://plants.ensembl.org, accessed on 8 May 2023). The hidden Markov model (HMM) file corresponding to the C3H domain (PF00642) was downloaded from the Pfam protein family database (http://pfam.xfam.org/, accessed on 8 May 2023). After inputting the HMM file and potato protein sequence file of C3H, we obtained 120 candidate potato *C3H* genes in Simple HMM Search [39]. All candidate genes that may have contained the C3H domain based on HMMER results were further examined by confirming the existence of the C3H core sequences using Pfam (https://www.ebi.ac.uk/Tools/pfa/pfamscan/, accessed on 9 May 2023), NCBI’s Conserved Domain Search (CD Search) (https://www.ncbi.nlm.nih.gov/Structure/bwrpsb/bwrpsb.cgi, accessed on 9 May 2023), and SMART (https://smart.embl.de/, accessed on 9 May 2023). Each potential gene was manually examined to ensure that C3H motifs were present. Fifty potato *C3H* gene models were finally identified in the potato genome after comprehensive curation. Using the ExPasy website (http://www.expasy.org/protparam/, accessed on 11 May 2023), we predicted and analyzed the physical and chemical properties of all potato C3H proteins [71]. The subcellular location predication of the identified potato C3H proteins was obtained by using tools from the WoLF PSORT Prediction website (https://wolfpsort.hgc.jp, accessed on 11 May 2023).

### 4.3. Location Analysis of C3H Gene Family Members on the Chromosome in Potato

The length information of 12 potato chromosomes and position information of potato *C3H* genes on the chromosome were obtained in the gff3 file, and we then used TBtools v1.120 to draw the *StC3H* gene location map and demonstrate the position information and distance relationship of the *StC3H* gene family members on the chromosome [39].

### 4.4. Multiple Sequence Alignment and Phylogenetic Analysis of C3H Gene Family in Potato

To study the evolutionary relationship of *C3H* genes, multiple alignments of amino acid sequences of full-length C3H proteins were performed using Muscle in MEGA 11 [72], and the phylogenetic trees were generated using the Neighbor-Joining (NJ) method in MEGA 11, with the following parameters: Poisson model, partial deletion (Site Cover Cutoff 90%), and 1000 bootstrap replications [73]. Sequences of C3H proteins from *Arabidopsis* were downloaded from the Plant Transcription Factor Database (http://planttfdb.gao-lab.org/, accessed on 16 May 2023), sequences of C3H proteins from tomatoes were downloaded from the Sol Genomics Network database (https://solgenomics.net, accessed on 16 May 2023). The phylogenetic tree was edited for visualization purposes with the online software EvolView (http://www.evolgenius.info/evolview/#login, accessed on 2 August 2023).

### 4.5. Analysis of Gene and Protein Structure and Function Prection of C3H Family in Potato

The gff3 file was used to extract the intron–exon and UTR (non-coding region) distribution and other gene structure information, which was finally visualized using TBtools. In order to better determine the distribution of conserved motifs in StC3H proteins, the online program Multiple Expectation Maximization for Motif Elicitation (MEME) was used (https://meme-suite.org/, accessed on 18 May 2023). The optimized parameters were employed as follows: maximum number of motifs, 10; optimum width of each motif, between 6 and 50 residues; other parameters were default values [74]. The homology-based three-dimensional protein structures were predicted using SWISS-MODEL Interactive Workshop (https://swissmodel.expasy.org/interactive, accessed on 3 August 2023), and we used the iDRBP_MMC website (http://bliulab.net/iDRBP_MMC/server, accessed on 3 August 2023) to identify DNA-binding proteins, RNA-binding proteins, and DNA/RNA-binding proteins [75,76].

### 4.6. Collinearity Analysis of StC3H Genes in Potato

We used Circos to map all *StC3H* genes onto potato chromosomes based on physical location information from the Potato Genome Sequencing Consortium (PGSC) database (http://solanaceae.plantbiology.msu.edu/, accessed on 5 June 2023) [77]. The Multiple Collinearity Scan toolkit (MCScanX) was used to analyze the gene duplication events, with the default parameters [38]. To exhibit the synteny relationship of the orthologous *C3H* genes obtained from potato and other selected species, the syntenic analysis maps were constructed using the Dual Systeny Plotter software (MCScanX) [39]. We downloaded the DNA files and gff3 files of the three plants from the potato genome database (http://solanaceae.plantbiology.msu.edu/, accessed on 8 May 2023), the *Arabidopsis thaliana* genome database (https://www.arabidopsis.org/, accessed on 16 May 2023), and the tomato genome database (https://solgenomics.net, accessed on 16 May 2023). We used the TBtools v1.120 to map the collinearity of the potato, *Arabidopsis*, and tomato genes, and the collinearity of *C3H* genes was labeled.

### 4.7. Cis-Acting Elements Analysis

To study the cis-acting elements in the promoter region of the *StC3H* gene, we retrieved the sequence of 2000 bp before the start codon of the *C3H* gene from the potato genome database and submitted it to the PlantCARE website (http://bioinformatics.psb.ugent.be/webtools/plantcare/html/, accessed on 20 May 2023) to predict cis-acting elements [78]. We then used TBtools v1.120 to plot the distribution of cis-acting components in the promoter region.

### 4.8. Expression of StC3H Genes in Different Tissues and Its Response to Different Stress

According to the potato transcriptome sequencing data downloaded from the PGSC website (http://solanaceae.plantbiology.msu.edu/, accessed on 10 June 2023), the expression data of *StC3H* under three kinds of abiotic stress, namely salt (NaCl, 150 mmol L^−1^, 24 h), drought (Mannitol, 260 μmol L^−1^, 24 h), and high temperature (35 °C, 24 h); biotic stress (Phytophthora infestans infected leaves (PIL)); and an immune inducer, including β-aminobutyric acid (BABA) and benzothiadiazole (BTH), were used to generate the *StC3H* expression heat map. In addition, the expression data of *StC3H* in different tissues (leaves, roots, petioles, stolons, flowers, young tubers, mature tubers, and stems) were collected, and then Hiplop Pro (https://hiplot.com.cn/, accessed on 21 June 2023) was used to standardize values and generate heat maps.

### 4.9. Total RNA Extraction and RT-qPCR

The total RNA from different samples was extracted using a SteadyPure Plant RNA Extraction Kit (Accurate Biology, Changsha, China). We then used a PrimeScript RT Reagent Kit (TakaRa, Kusatsu, Japan) to generate cDNA following the instructions of the kit. The Real-Time PCR program was performed as follows: (1) 95 °C for 2 min (preincubation); (2) 95 °C for 15 s; and (3) 60 °C for 20 s, repeated 40 times. The remaining steps used the instrument’s default settings. The *E*F_1_*a* gene was used as a reference gene. Three independent biological replicates were examined, and the gene expression levels were calculated as 2^−∆∆Ct^. The primers for RT-qPCR are listed in Table S4.

## 5. Conclusions

Despite the diverse roles of *C3H* genes gradually revealed in different plants, the functions of *C3H* genes in potato have not yet been well elucidated. In this study, we identified 50 *C3H* genes in potato, and characterized the phylogenetic relationships, gene structures, physical and chemical properties, and expansion patterns of these *StC3H* genes. The results disclosed that all 50 *StC3H* genes were classified into 10 groups, and segmental duplications were essential for the extension of the *StC3H* family. Structure and function prediction analysis showed that most of the StC3H proteins could interact with RNA or DNA. According to their expression profiles, it could be concluded that *StC3H* genes are involved in potato growth and development and diverse stress responses. Furthermore, RT-qPCR data showed that many *StC3H* genes were significantly induced by heat stress. Tissue-specific expression showed that the *StC3H* genes expressed varied widely in the potato tissues, especially in the stolon and developing tubers, indicating that *StC3H* genes may participate in tuber development. Overall, these results provide a reference for further revealing the function of the *StC3H* genes and breeding new potato varieties with strong adaptability and good stress resistance.

## Figures and Tables

**Table 1:** The *C3H* genes in potato and properties of the deduced proteins (*Solanum tubersom*).

Gene Name	Gene ID	Protein				Subcellular Location
		Number of Amino Acids (aa)	Molecular Weight (Da)	Theoretical pI	Grand Average of Hydropathicity	
StC3H-1	PGSC0003DMG400016400	707	79,152.04	5.80	−1.060	Nucleus
StC3H-2	PGSC0003DMG400010493	650	71,922.33	6.00	−0.570	Nucleus
StC3H-3	PGSC0003DMG400024054	223	23,792.81	9.01	−0.480	Nucleus
StC3H-4	PGSC0003DMG400022651	492	51,506.80	8.41	−0.390	Nucleus
StC3H-5	PGSC0003DMG400006828	739	80,448.74	5.93	−0.503	Nucleus
StC3H-6	PGSC0003DMG400006803	730	79,277.70	6.02	−0.468	Nucleus
StC3H-7	PGSC0003DMG400006330	289	30,656.69	9.33	−0.438	Nucleus
StC3H-8	PGSC0003DMG400002748	129	14,562.29	8.61	−0.888	Cytoplasm
StC3H-9	PGSC0003DMG400020705	761	89,133.84	6.62	−1.436	Peroxisome
StC3H-10	PGSC0003DMG400001658	702	77,141.25	6.67	−0.512	Nucleus
StC3H-11	PGSC0003DMG400010434	477	53,542.39	5.78	−0.882	Nucleus
StC3H-12	PGSC0003DMG400013655	338	38,113.47	7.12	−1.036	Nucleus
StC3H-13	PGSC0003DMG400003622	858	91,533.33	5.48	−0.722	Nucleus
StC3H-14	PGSC0003DMG400030591	341	38,323.55	6.02	−0.748	Nucleus
StC3H-15	PGSC0003DMG400009821	284	30,014.31	9.57	−0.381	Cytoplasm
StC3H-16	PGSC0003DMG400014305	430	47,162.96	7.88	−0.374	Chloroplast
StC3H-17	PGSC0003DMG400026471	349	38,383.75	6.19	−0.690	Nucleus
StC3H-18	PGSC0003DMG400015168	518	58,326.50	5.14	−1.168	Nucleus
StC3H-19	PGSC0003DMG400000604	224	24,991.78	8.80	−0.724	Nucleus
StC3H-20	PGSC0003DMG400038576	212	24,095.85	6.14	−0.979	Nucleus
StC3H-21	PGSC0003DMG400005636	352	41,316.02	8.51	−1.540	Nucleus
StC3H-22	PGSC0003DMG400031374	708	79,259.80	5.93	−1.084	Nucleus
StC3H-23	PGSC0003DMG402025318	159	17,809.09	6.43	−0.834	Chloroplast
StC3H-24	PGSC0003DMG400024801	858	96,138.64	9.10	−1.114	Nucleus
StC3H-25	PGSC0003DMG400014565	832	91,063.68	6.40	−0.707	Nucleus
StC3H-26	PGSC0003DMG400030567	338	37,972.62	7.72	−0.684	Nucleus
StC3H-27	PGSC0003DMG400005333	397	43,110.49	8.51	−0.455	Nucleus
StC3H-28	PGSC0003DMG400030758	396	42,133.40	6.10	−0.380	Nucleus
StC3H-29	PGSC0003DMG402027067	603	67,135.00	5.15	−0.862	Nucleus
StC3H-30	PGSC0003DMG400026937	357	39,088.35	6.79	−0.743	Nucleus
StC3H-31	PGSC0003DMG400030361	668	73,112.19	5.97	−0.434	Nucleus
StC3H-32	PGSC0003DMG400032829	427	47,977.30	7.59	−0.725	Nucleus
StC3H-33	PGSC0003DMG400025459	350	39,389.36	6.46	−0.579	Nucleus
StC3H-34	PGSC0003DMG400014784	495	55,654.30	5.53	−0.779	Nucleus
StC3H-35	PGSC0003DMG400011383	435	46,976.53	8.81	−0.315	Nucleus
StC3H-36	PGSC0003DMG400024171	336	36,065.91	7.59	−0.534	Nucleus
StC3H-37	PGSC0003DMG400023710	294	30,815.82	9.30	−0.499	Cytoplasm
StC3H-38	PGSC0003DMG400023719	697	76,264.47	6.83	−0.478	Nucleus
StC3H-39	PGSC0003DMG400008150	701	76,411.82	6.61	−0.444	Nucleus
StC3H-40	PGSC0003DMG400011414	301	31,381.47	9.35	−0.503	Chloroplast
StC3H-41	PGSC0003DMG400016236	433	47,084.25	8.85	−0.463	Nucleus
StC3H-42	PGSC0003DMG400029993	701	76,760.82	5.82	−0.489	Nucleus
StC3H-43	PGSC0003DMG400026524	331	38,988.95	9.62	−1.358	Nucleus
StC3H-44	PGSC0003DMG400025515	335	38,362.12	6.76	−0.885	Nucleus
StC3H-45	PGSC0003DMG400002893	425	45,207.41	8.58	−0.687	Nucleus
StC3H-46	PGSC0003DMG400000350	372	42,312.18	5.84	−0.815	Nucleus
StC3H-47	PGSC0003DMG400046840	215	24,845.06	6.50	−0.899	Cytoplasm
StC3H-48	PGSC0003DMG400005394	385	43,943.14	6.08	−0.497	Cytoskeleton
StC3H-49	PGSC0003DMG400045292	414	47,260.84	6.08	−0.710	Nucleus
StC3H-50	PGSC0003DMG400043565	281	32,097.45	6.56	−0.702	Cytoskeleton

## Data Availability

Not applicable.

## Supplementary Materials

The following supporting information can be downloaded at: https://www.mdpi.com/article/10.3390/ijms241612888/s1.
