# Supplementary material for: Genome-Wide Identification and Expression Analysis of C3H Zinc Finger Family in Potato (Solanum tuberosum L.)
Source: Int J Mol Sci. 2023 Aug 17;24(16):12888. doi: 10.3390/ijms241612888 (PMC10454627; doi:10.3390/ijms241612888)
Supplement: Supplementary file 1 [file ijms-24-12888-s001.zip › Supplementary Figuere Data Sheet 1.pdf]

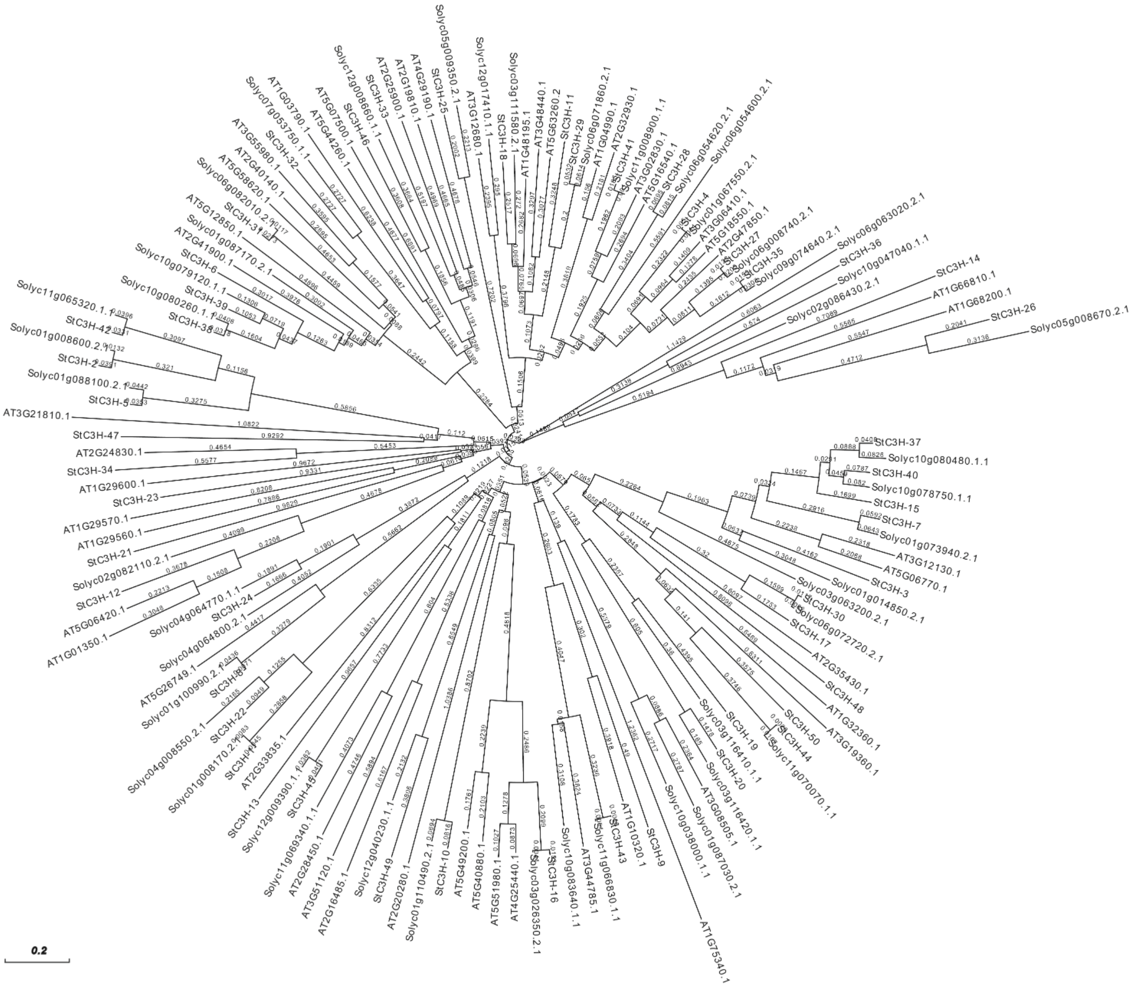

Figure S1. Phylogenetic tree of C3H genes in potato, Arabidopsis and tomato. The value on the branches of the phylogenetic tree represent a phylogenetic relationship between genes. The scale bar corresponds to 0.2 estimated amino acid substitutions per site.

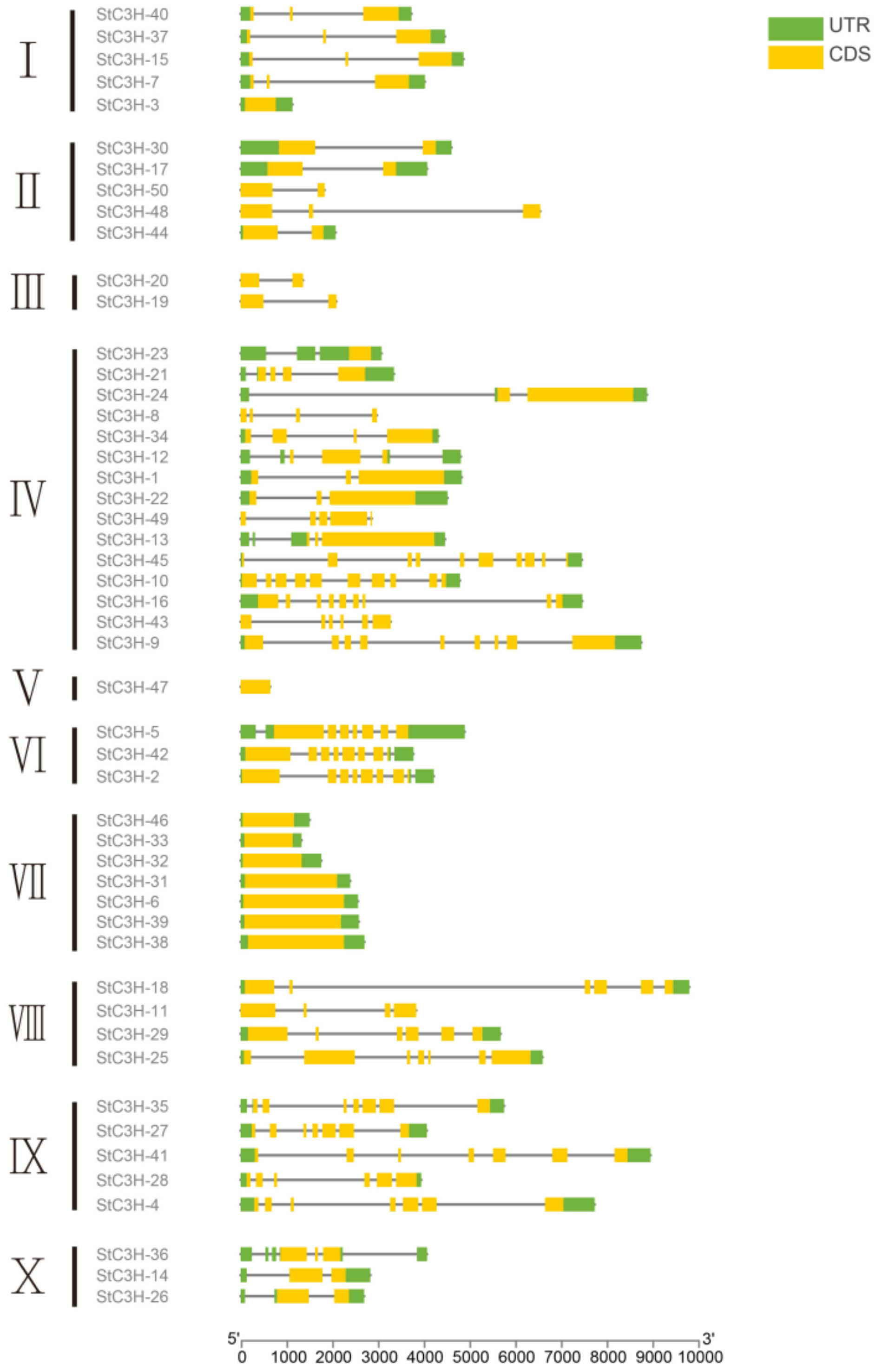

Figure S2. Structure of *C3H* genes in potato. Green boxes indicate untranslated regions; yellow boxes indicate exons; black lines indicate introns.

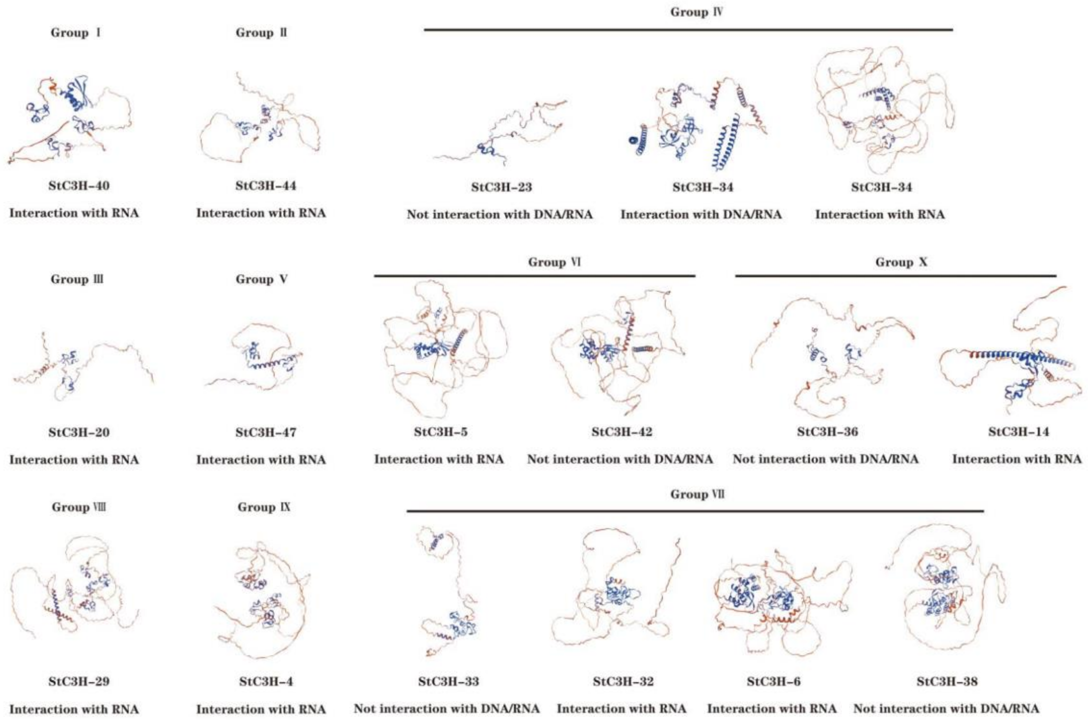

Figure S3. The 3D models of the StC3H proteins in different groups. Representative protein structures in different groups were displayed. The blue colour indicates high confidence, and the orange colour indicates low confidence
