# Supplementary figures and images for: Genome-Wide Identification and Expression Analysis of C3H Zinc Finger Family in Potato (Solanum tuberosum L.)
Source: Int J Mol Sci. 2023 Aug 17;24(16):12888. doi: 10.3390/ijms241612888 (PMC10454627; doi:10.3390/ijms241612888)

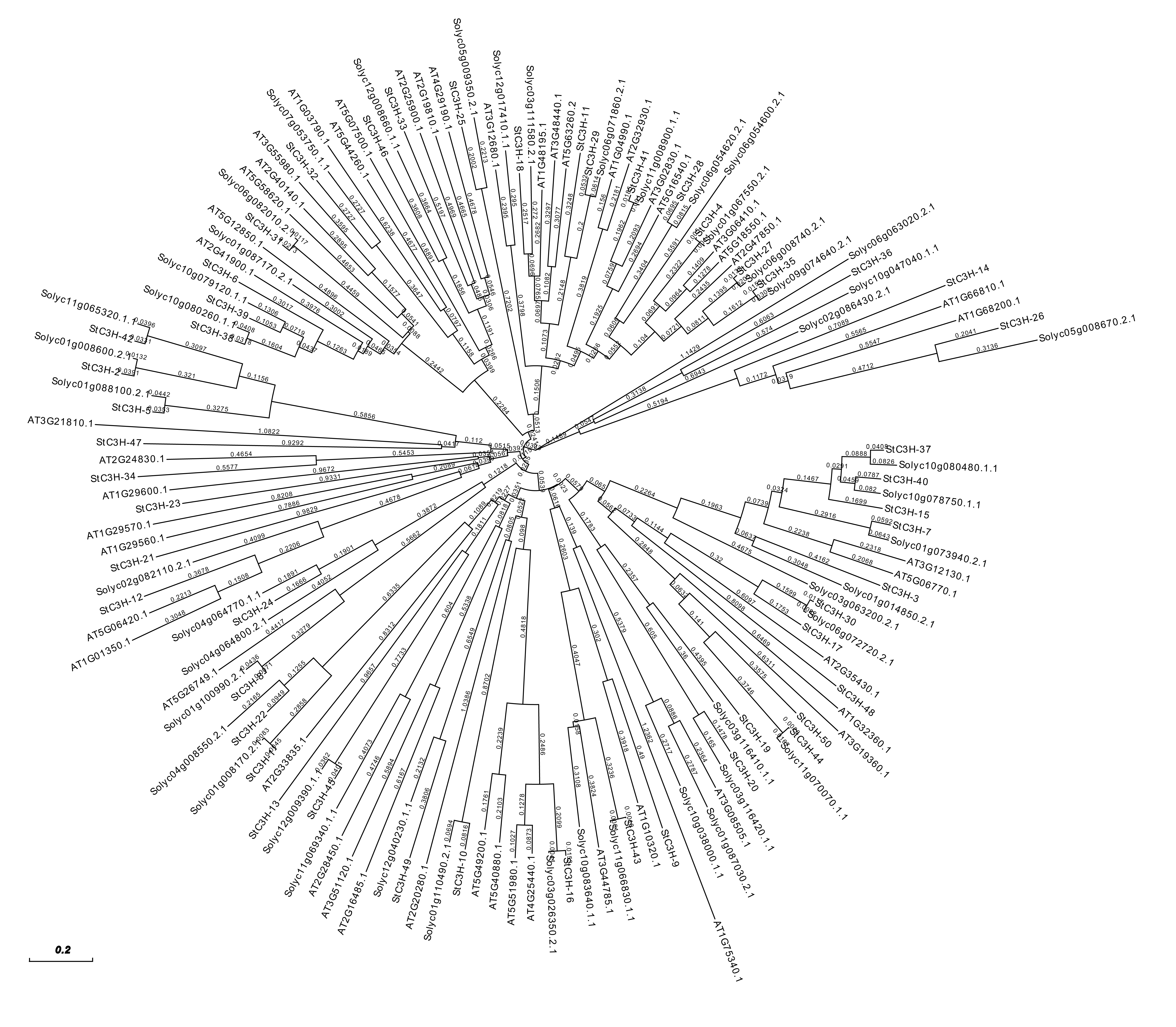

Supplement: Supplementary file 1 [file ijms-24-12888-s001.zip › Figure S1.jpg]

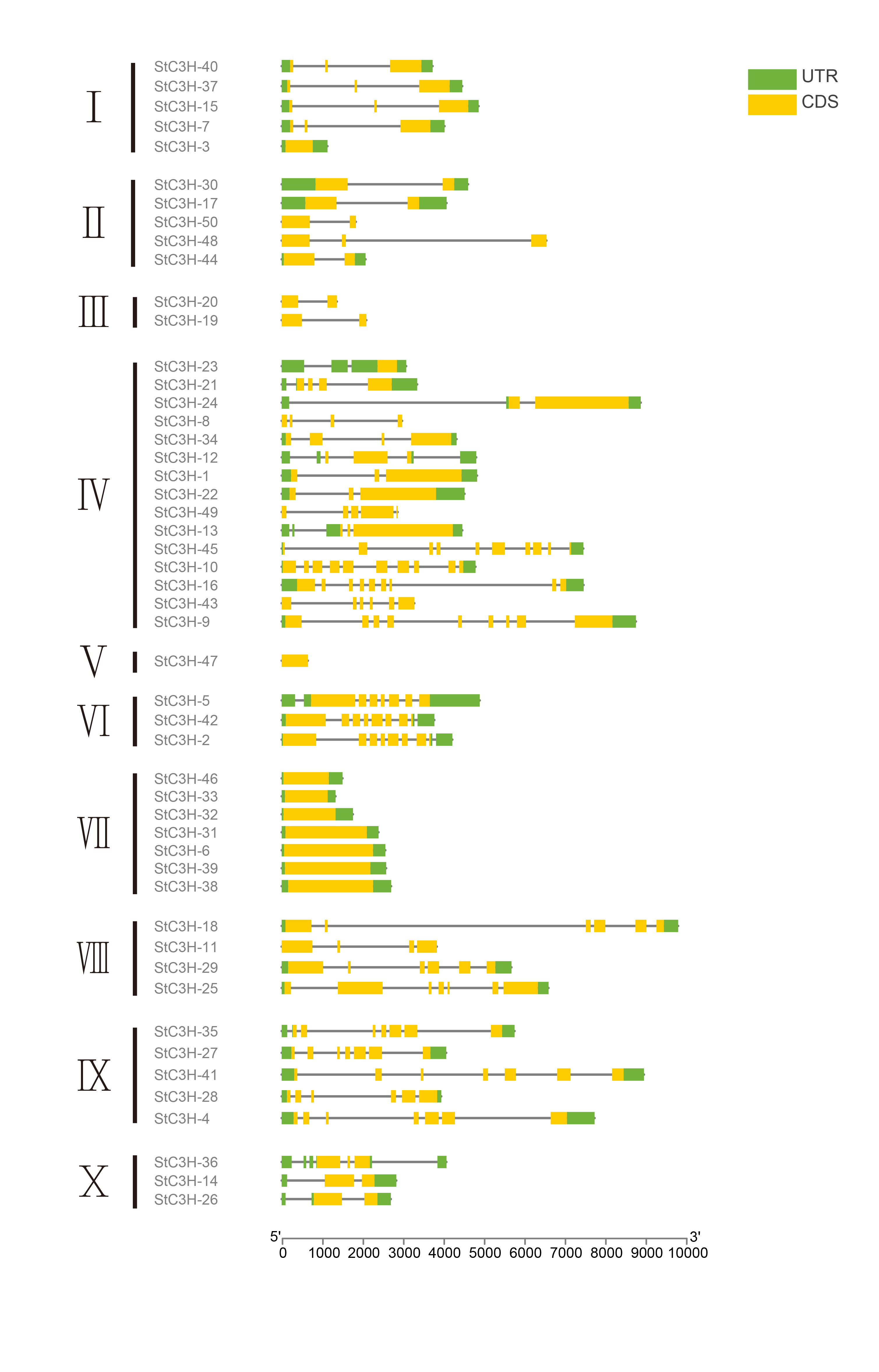

Supplement: Supplementary file 1 [file ijms-24-12888-s001.zip › Figure S2.jpg]

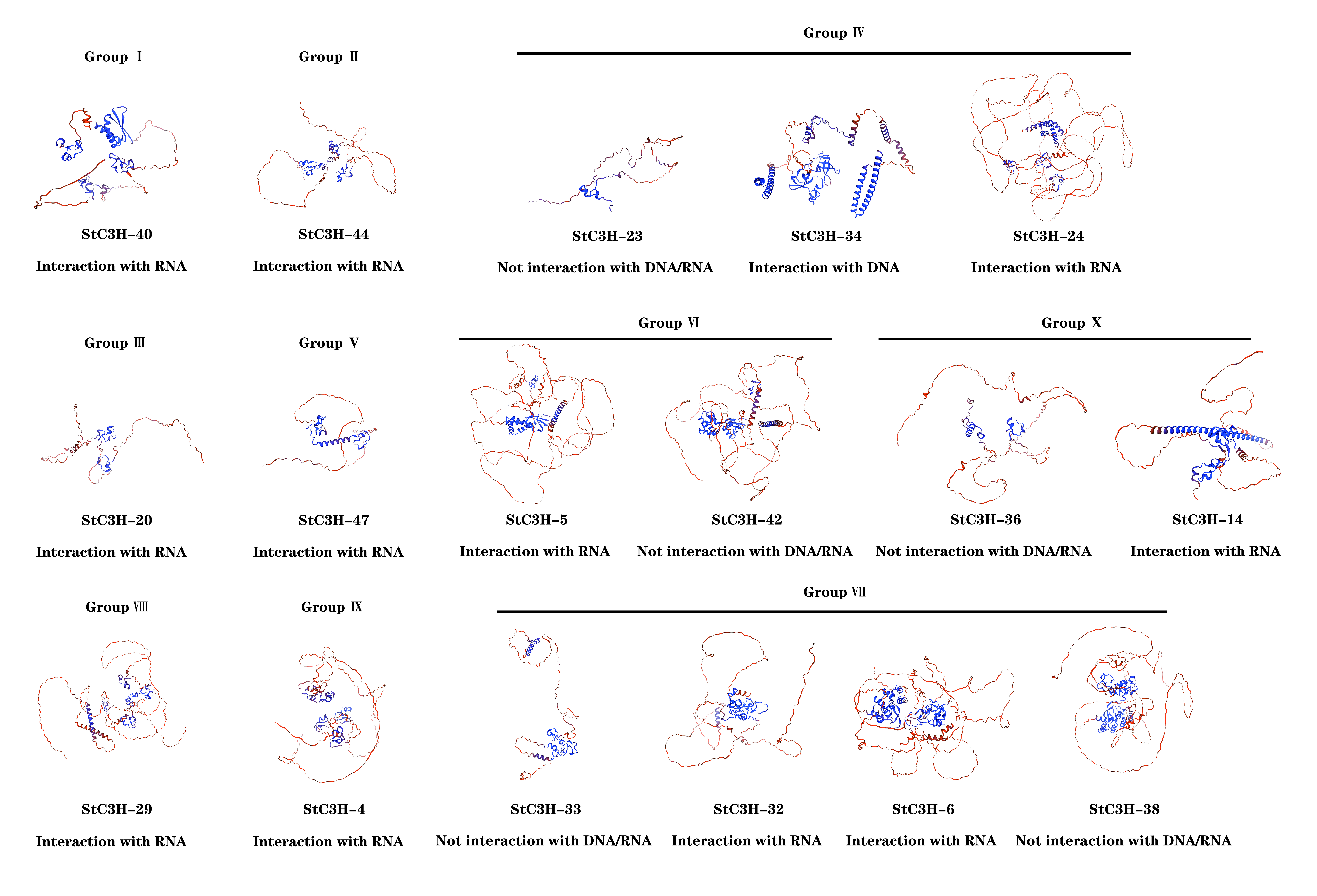

Supplement: Supplementary file 1 [file ijms-24-12888-s001.zip › Figure S3.jpg]
